# Effects of Fecal Occult Blood Immunoassay Screening for Colorectal Cancer—Experience from a Hospital in Central Taiwan

**DOI:** 10.3390/medicina59040680

**Published:** 2023-03-29

**Authors:** Pei-Yu Yang, I-Ting Yang, Tzu-Hsuan Chiang, Chi-Hong Tsai, Yu-Ying Yang, I-Ching Lin

**Affiliations:** 1 Department of Laboratory, Show-Chwan Memorial Hospital, No. 542, Sec1, Chung-Shan Rd., Changhua 500, Taiwan; a2900@show.org.tw (P.-Y.Y.); a11621@show.org.tw (I.-T.Y.); a12793@show.org.tw (T.-H.C.); al009@show.org.tw (Y.-Y.Y.); 2 Department of Kinesiology, Health and Leisure, Chienkuo Technology University, No. 1, Chiehshou North Road, Changhua 500, Taiwan; 3 Department of Surgery, Show-Chwan Memorial Hospital, No. 542, Sec1, Chung-Shan Rd., Changhua 500, Taiwan; ads031@show.org.tw; 4 Department of Family Medicine, Asia University Hospital, No. 222, Fuxin Rd., Wufeng Dist., Taichung 41354, Taiwan; 5 Department of Healthcare Administration, Asia University, No. 500, Lioufeng Rd., Wufeng Dist., Taichung 41354, Taiwan

**Keywords:** colorectal cancer, stool occult blood immunoassay, fecal immunochemical test

## Abstract

*Background and Objectives*: In 2004, the Health Administration of Taiwan began to promote a hospital-based cancer screening quality improvement program, under the principle that “prevention is better than therapy”. The aim of this study was to evaluate the effectiveness of colorectal cancer (CRC) screening in patients who received a fecal immunochemical test (FIT) at a hospital in central Taiwan. *Materials and Methods*: This was a retrospective study. *Results*: Fecal occult blood immunoassays for CRC screening were conducted in 58,891 participants, of whom 6533 were positive (positive detection rate 11.10%). The positive patients then underwent colonoscopy, and the detection rates of polyps and CRC accounted for 53.6% and 2.4% of all colonoscopy-confirmed diagnoses (3607), respectively. We further enrolled data from patients diagnosed with CRC at our hospital from 2010 to 2018. The patients with CRC were divided into two groups according to whether or not they had received fecal occult blood screening. Among the 88 patients with CRC by screening, 54 had detailed medical records including cancer stage. Of these 54 patients, 1 (1.8%) had pre-stage, 11 (20.4%) had stage I, 24 (44.4%) had stage II, 10 (18.5%) had stage III, and 8 (14.8%) had stage IV CRC. The early cancer detection rates of the screening and non-screening groups were 66.7% and 52.7%, respectively, and the difference was significant (*p* = 0.00130). *Conclusions*: In this study, screening with FIT significantly increased the early detection of CRC. The main advantage of FIT is the non-invasiveness and low cost. It is hoped that the further adoption of early screening can increase the detection rates of colorectal polyps or early cancer to improve survival, reduce the high cost of subsequent cancer treatment, and reduce the burden on the patient and healthcare system.

## 1. Introduction

Cancer is a very important public health problem worldwide. According to the World Health Organization (WHO), cancer caused about 10 million deaths in 2020. Colorectal cancer (CRC) is the third most common cancer in men and the second most common cancer in women. More than 1.93 million new cases of CRC were reported in 2020, and about 920,000 people died from CRC-related diseases [1]. In Taiwan, according to the “109-Year Statistical Analysis of Causes of Death” published by the Ministry of Health and Welfare, cancer has ranked first among the top ten causes of death since 1971. 

Due to the increasing Westernization of diet and the aging population, Asian countries have experienced a marked increase in CRC incidence and mortality over the past few decades. In Taiwan, the CRC mortality rate is 14.0–15.0 per 100,000 people, which has a great impact on the healthcare system. Statistics from the Health Promotion Administration (HPA) show that the peak incidence of CRC occurs in people older than 50 years, and then gradually slows after 80 years of age. According to the Ministry of Health and Welfare, from 2004 to 2008, the 5-year survival rates of pre-stage colorectal, stage I, stage II, and stage III cancer were 86%, 81%, 72%, and 57%, respectively. Moreover, the 5-year survival rate of stage IV was only 12%, so early screening is very important. The HPA has provided fecal occult blood screening for CRC to residents aged 50–69 years since 2004. Every year, more than 1 million citizens are eligible and undergo the screening. In 2013, the age was expanded to 74 years. Residents who meet the screening conditions go to a medical institution or health center, and screening is performed every 2 years. If the screening result is positive, a colonoscopy examination is recommended for further confirmation [2,3].

The fecal occult blood test (FOBT) is the most widely used diagnostic test for CRC. The FOBT detects blood or blood products in stool via two main approaches: chemical, or immunological (fecal immunochemical test, FIT). Screening using the chemical FOBT has been shown to reduce CRC mortality in large randomized controlled trials [4]. However, FIT has a specific response to human fecal hemoglobin, albumin, or other fecal blood components, and therefore it does not require dietary or drug restrictions, which increases public participation. The biggest advantage is its ability to detect and quantify fecal hemoglobin concentration, with 7 to 15 times greater sensitivity compared with chemical tests. In addition, the accurate automated analysis of the FIT can prevent personal identification. Due to these advantages, FIT is used for large-scale screening programs for CRC in various countries.

The aim of this study was to evaluate the effectiveness of CRC screening in patients who received a FIT through the HPA screening program at a hospital in central Taiwan. We also collected data from patients diagnosed with CRC at our hospital from 2010 to 2018, and compared those who did and did not receive an FOBT.

## 2. Materials and Methods

### 2.1. Research Subjects

The HPA implemented a four-cancer screening program in 2004. This study retrospectively traced those who were screened in this program at a regional hospital in central Taiwan from 2010 to 2018. The information of the subjects who received screening included basic information such as gender, age, and education level. In addition, information on health behaviors includes the amount of weekly exercise, the consumption of at least 1.5 bowls of vegetables and 2.5 servings of fruit every day, and a family history of CRC.

In addition, CRC patients diagnosed at our hospital from 2010 to 2018 were also collected from the cancer registration system of our hospital, and were divided into two groups: those with CRC detected by fecal occult blood screening and those without CRC detected by screening. 

### 2.2. Fecal Occult Blood Screening

A Kyowa HMJACK system (Minaris Medical Co., Ltd., Osaka, Japan) was used for the screening tests. The tested hemoglobin cut-off value was 8 ng/mL, equivalent to 20 μg hemoglobin per gram of feces, and a test result above the cut-off value was defined as being positive; test results were reported to the participants by mail or telephone. Participants with positive results were then recommended for referral for a complete colonoscopy or sigmoidoscopy with a barium enema for confirmation.

### 2.3. Statistics

Statistical analysis was performed using SPSS version 26 (IBM Inc., Armonk, NY, USA). *p*-value less than 0.05 was considered significant.

## 3. Results

From 2010 to 2018, a total of 58,891 people received CRC screening at our hospital, of whom 29,732 (50.5%) were male and 29,159 (49.5%) were female, and the average age was 60.08 ± 6.91 years (Table 1). The average annual number of participants tested was 6544, and the average annual positive rate of fecal occult blood immunoassay was 11.3%. When participating in the screening program, the participants were asked to complete a questionnaire including basic information and health behavior.

### 3.1. Association between Basic Information of the Participants and the Statistical Analysis of the FIT Screening Results

Table 2 shows the basic information of the participants and the statistical analysis of the FIT screening results. There was no significant sex difference in the number of subjects tested. Those aged 50–60 years accounted for the largest proportion of those tested (53.4%), followed by those aged 61–70 years (36.3%), and those aged over 75 years accounted for the smallest proportion (10.3%). The positive screening test rate was 13.0% for males and 9.6% for females, including 12.8% and 17.8% for those over 70 years of age. An education level of elementary school accounted for the largest proportion of those tested (30%), followed by high school (26%). The positive rate was highest for the uneducated (14.9%), and lowest for those with an education level of graduate school and above (6.4%).

### 3.2. Association between Participants’ Health Behaviors and the Statistical Analysis of the FIT Screening Result

Table 3 shows the statistical analysis of the participants’ health behaviors and FIT screening results. Overall, 33.6% of the subjects exercised more than three times a week, and the positive screening test rate in these subjects was 9.7%, compared to 11.8% in those who exercised less than three times a week. In addition, 79.8% of the participants ate at least 1.5 bowls of vegetables and 2.5 servings of fruit every day, and the positive rate in these subjects was 10.1%, compared to 11.3% in those who did not consume these levels. The positive rate in those with a family history of CRC was 10.8%, compared to 11.3% in those without a family history.

### 3.3. Confirmed Diagnosis of FIT Positive Results

A total of 3607 patients had positive screening results and colonoscopy confirmed diagnoses, including 2045 males (56.7%) and 1562 females (43.3%) with an average age of 61.4 ± 7.0 years (range 50–75 years). Hemorrhoids and polyps accounted for 31.3% and 53.6% of the diagnoses, respectively, and 2.4% were diagnosed with CRC (Table 4).

According to the data collected from the hospital’s cancer registration system from 2010 to 2018, there were 900 patients with CRC (535 males (59.4%) and 365 females (40.5%)). Fifty-four cases were confirmed by FIT screening and had complete information, of whom 37 were male (68.5%), and 17 were female (31.5%).

### 3.4. The Basic Information and Health Behaviors of the Subjects Were Analyzed Using Logistic Regression

The basic information and health behaviors of the subjects were analyzed using logistic regression (Table 5 and Table 6). The results of the basic information analysis showed that the probability of a positive screening result in the males was 1.455 times that of the females (*p* < 0.001). The risk of a positive screening result in those aged 50–60 years was 0.563 times (*p* < 0.001) that of those aged 61–74 years, and the risk of a positive screening result in those aged 61–70 years was 0.714 times that of those aged 71–74 years (*p* < 0.001). The risk of a positive screening result in those who were uneducated was 0.734 times that of those with junior high school education (*p* < 0.001), 0.568 times that of those with high school education (*p* < 0.001), and 0.577 times that of those with a college education (*p* < 0.001), but 0.466 times that of those with a graduate degree or above (*p* < 0.001). With regards to health behaviors, the risk of a positive screening result in those who did not eat at least 1.5 bowls of vegetables and two servings of fruits per day was 1.427 times that of those who did eat these levels (*p* < 0.001).

### 3.5. The Different Results of CRC Stage in Screening and non-Screening Group

According to the data collected from the cancer registration system at our hospital from 2010 to 2018, there were 900 CRC patients, including 535 males (59.4%) and 365 females (40.5%), and the clinical staging records of 733 patients were complete. Among these patients, 54 were found to have CRC by FIT screening, and 679 were found to have CRC clinically (non-screening) (Table 7). The average age of the screening group was 61.2 ± 6.4 years, and the average age of the non-screening group was 67.8 ± 13.6 years. Further analysis of CRC stages found that the discovery rate in those with pre-stage, stage I, and stage II CRC was significantly higher in the screening group (68.3%) than in the non-screening group (52.2%).

## 4. Discussion

In the past few years, the cost of CRC treatment has increased rapidly. From 1990 to 2003, according to the degree of disease at the time of diagnosis, the cost of treatment per case increased by about 200%, while the cost of screening did not increase. Screening to prevent end-stage CRC and death has resulted in significant savings. Screening for CRC involves various approaches, policies, and interventions, such as FOBT annually, sigmoidoscopy every 5 years, FOBT annually and sigmoidoscopy every 5 years, barium enema every 5 years, or sigmoidoscopy every 10 years; these methods can be used in combination or alone. A systematic analysis by Khalili et al. in 2020 showed that any strategy for CRC screening was more cost-effective compared to a non-screening approach [5]. Using FIT to detect fecal hemoglobin is currently the best approach, and it is widely used to screen for CRC. It is non-invasive and inexpensive. The Taiwan government started a CRC screening program in 2004 as part of the cancer prevention and control plan. From 2004 to 2009, residents aged 50–69 were eligible for FIT screening every 2 years, and if tumors were detected, further interventional treatment would be given. In symptomatic patients, a UK primary care study reported a sensitivity and specificity of 91% for CRC when FIT results were ≥10 μg/g [6]. 

This study collected 58,891 subjects who participated in CRC screening at our hospital from 2010 to 2018 The number of participants and positive screening results are listed in Table 1. The average number of participants was about 6500 per year, and the positive screening rate was 11.3%. The positive rate at our hospital is higher than that published by Chiu et al. In a meta-analysis, Saw reported that when using the HM-JACKarc test and using 10 μg Hb as the positive threshold, the positive rate ranged from 16 to 22.1%. In 2018, the positive rate in Shanghai was reported to be 10.9% [1] and 17.5% in Guangzhou [2], and Almansoori et al. reported that the positive rate was 13.5% in Dubai. The positive rate varies depending on the region and age distribution of those tested, sampling ambient temperature, and transportation and storage before analysis [7]. Table 2 shows the basic information of the subjects and the statistical analysis of the FIT screening results. The positive rate in males was greater than in females, and the younger was less than the elderly. With regards to education level, most of the participants (30%) had a level of elementary school, followed by high school (26%). Elementary school education has encompassed 9 years of national education since 1968 in Taiwan. Therefore, most of those over the age of 60 years received basic elementary school education. The positive rate was highest (14.9%) for the uneducated, and lowest (6.4%) for those with a graduate degree or above. Taken together, the risk of a positive screening test was significantly lower in those with a higher education level, and the risk was also lower in those who were younger and female. These results are consistent with other studies [2,3,4]. It is possible that a higher level of knowledge has a great impact on the risk of disease, and that it may increase participation in regular physical examinations [6].

Table 3 shows the statistical analysis of the participants’ health behaviors and FIT screening results. Overall, 33.6% of the participants exercised more than three times a week. The positive rate was 9.7% in this group compared to 11.8% in those who exercised less than three times a week, and the difference was significant (*p* = 0.02). In addition, 79.8% of the participants ate at least 1.5 bowls of vegetables and 2.5 servings of fruit every day. The positive screening rate was 10.1% in this group, compared to 11.3% in those who did not consume these levels. In a meta-analysis of 756,217 participants with 6–20 years of follow-up, Baena et al. concluded that fruit and vegetable intake was associated with a modest reduction in CRC risk by 9%. [7]. With regards to family history, the positive rate in those with a family history of CRC was 10.8%, compared to 11.3% in those without a family history. Family history is highly correlated with cancer. We speculate that the possible reason for the inconsistency in the results is under-reporting. We found that the highly educated answered, first-degree relatives (FDR), and they had the highest proportion of CRC or other cancers (10.5%, 35.9%), and that those who were uneducated had the lowest rate (2.8%, 9.0%). In addition, the proportion of FDRs with CRC was lower with increasing age (3.7% in those 75 years of age), and highest (6.0%) in those aged 50–60 years. In 1999, Glanz reported that more than a quarter of respondents who were known to have siblings or parents with CRC reported no FDR with CRC [5,6,7,8,9,10,11]. In addition, Caucasian, male, less educated, and older respondents were often unaware that the underreporting of CRC family history could be due to confusion or lack of awareness of cancer in relatives. Many studies have shown large differences in the accuracy of reporting family history by cancer type, degree of association, education level, and gender. Clinically, failure to report a family cancer history may lead to missed opportunities for surveillance, and ultimately avoidable mortality [5,6] Therefore, clear and accurate communication between doctors, patients, and relatives is very important. Healthcare providers should emphasize the need for open family communication among patients with CRC, and emphasize that health information is important for other family members to make health decisions. The accuracy of family health information is critical to the advancement of research, clinical services, counseling, and patient education. Previous studies have indicated that individuals with relatives who have had CRC are at a higher risk compared with the general population. However, the social stigma associated with bowel cancer affects the accuracy of reporting a family history of CRC [5,6,7,8,9,10,11], as also reported in southern Taiwan [12]. However, many studies have shown that modifiable lifestyle factors including smoking, alcohol consumption, physical activity, and body mass index contribute more to CRC than family history [13,14]. 

We identified a total of 3608 participants with positive FIT results and follow-up confirmation examinations. Among the confirmed results, hemorrhoids (31.3%) and polyps (53.7%) were the major reasons, and 2.4% were diagnosed with CRC (Table 4). A total of 1936 patients diagnosed with polyps were screened at our hospital, most of whom were male (male-to-female ratio of 1.67:1). In 2017, Kim et al. reported that a considerable number of patients with hemorrhoids had positive FIT results (33.0%) [15]. In addition, Chiu et al. reported that about 4–10% of the screened subjects in an Asian population screening program had positive FIT results and needed further confirmatory examinations (colonoscopy or double-contrast barium enema) [3]. Our results are similar to these reports. 

The detection rates of polyps and CRC vary by country or region. For example, Almansoori et al. reported that polyps were found in 30.5% of confirmed cases in Dubai, of whom 0.2% had CRC [16]. Other studies include rates of 67% and 7% in Ireland [17], 55.3% and 3.7% in Canada [18], 47.3% and 0.9% in Singapore [19], and 32.2% and 1.6% in Guangzhou, China [2]. Most polyps are harmless, but some may become cancerous, and the larger the polyp, the greater the risk. Chiu et al. investigated the screening results of millions of people in Taiwan, from 2004 to 2009, and found that the rate of CRC accounted for 0.85% of the confirmed diagnoses. The results of CRC in the present study are higher than in Chiu et al.’s study. This may be because the year of screening and the age range of those screened were different (data were collected in this study from 2010 to 2018 and the age range of those screening was 50–74 years compared to 50–69 years in Chiu et al.’s study) [3].

Our results show that FIT screening increased the early-stage detection rate by about 16%, which is higher than that in outpatient clinics, and the difference between the two groups was significant (*p* = 0.0013). The results are similar to those reported by Chiu et al. [3]. In their study, the mortality rate of the screened group was 13.77 per 100,000, compared to 36.31 per 100,000 in the unscreened group. Hence, FIT screening reduced the CRC mortality rate by about 62%. If it can increase the screening rate from 21.4% to 60%, the mortality rate can be reduced by 36% and improve the quality of life. These findings may help convince health policymakers that it is worthwhile to continue promoting such a screening program with existing resources [3]. 

## 5. Conclusions

In this study, screening with FIT significantly increased the early detection of CRC. The main advantage of FIT is the non-invasiveness and low cost. It is hoped that the further adoption of early screening can increase the detection rates of colorectal polyps or early cancer to improve survival, reduce the high cost of subsequent cancer treatment, and reduce the burden on the patient and healthcare system.

### Research Limitations

We found that it was difficult to trace those with a positive FOBT, and there were 3231 people (329 people/year) who had still not received a further diagnosis, which shows that there is still room for improvement. The reasons for not receiving a confirmatory diagnosis included unknown reasons (*n* = 344), followed by a refusal to be diagnosed (*n* = 128). Other reasons included returning to the clinic more than 2 months after the diagnosis; hemorrhoids or polyps were diagnosed in the outpatient clinic; low willingness to return to the clinic; and physical discomfort (chronic disease) and so did not want to undergo an invasive examination. There is still a potential risk of CRC (polyps) in patients who have not received a diagnosis. However, studies in Hong Kong and Italy have shown that the main reasons for not participating in FOBT-based screening programs include a lack of time and fear that a positive test result will have a major impact on life. Therefore, it may be more beneficial to actively follow up the cases with positive fecal occult blood tests for further diagnosis as soon as possible, so as not to lose the opportunity of early detection and treatment and to consider the combination of different types of interventions.

## Figures and Tables

**Table 1:** The number of men and women in each year and the inspection results.

		Gender (No.)				Results	
Year	Male	Female	Total	Negative (No.)	Negative Rate(%)	Positive (No.)	Positive Rate (%)
2010	3852	3714	7566	6671	88.2%	761	11.8%
2011	2640	2414	5054	4467	88.4%	587	11.6%
2012	2681	2536	5217	4671	89.5%	546	10.5%
2013	3568	3318	6886	6050	87.9%	836	12.1%
2014	3401	3376	6777	5895	87.0%	882	13.0%
2015	3765	3542	7307	6472	88.6%	835	11.4%
2016	3306	3434	6740	6049	89.7%	691	10.3%
2017	3087	3130	6217	5547	89.2%	670	10.8%
2018	3432	3695	7127	6402	89.8%	725	10.2%

**Table 2:** Statistical analysis of basic information of participants and FOBT screening results.

Item	Tested Participants, (No.)	Results		
		Negative (%)	Positive (%)	*p*-Value
Total	58,891	52,224 (88.7%)	6667 (11.3%)	<0.01
Gender				
Male	29,732	25,873 (87.0%)	3859 (13.0%)	
Female	29,159	26,351 (90.4%)	2808 (9.6%)	
Age(year/old)				<0.01
50–60	31,432	28,577 (90.9%)	2855 (9.1%)	
61–70	21,386	18,653 (87.2%)	2733 (12.8%)	
>70	6073	4994 (82.2%)	1079 (17.8%)	
Education	25,696	22,984	2712	<0.01
None	1718	1462	256 (14.9%)	
Elementary school	7823	6753	1070 (13.7%)	
Junior high school	5177	4651	526 (10.2%)	
High school	6738	6206	532 (7.9%)	
College, University	3515	3229	286 (8.1%)	
Graduate school or above	640	599	41 (6.4%)	
Unanswered	33,280	29,324	3956	

**Table 3:** Statistical analysis of participants’ health behaviors and FOBT screening results.

Item	Tested Participants, (No.)	Results		
		Negative (%)	Positive (%)	*p* Value
**Total**	58,891	52,224 (88.7%)	6667 (11.3%)	<0.01
**Exercise times per week**	18,171			
0–2	14,368	12,666	1702 (11.8%)	
3–6	4187	3787	400 (9.6%)	
≧7	1910	1718	192 (10.1%)	
Unanswered	34,053			
**Every exercise period**				0.045
<30 min	3587	3186	401 (11.2%)	
30–60 min	6415	5817	598 (9.3%)	
1–2 h	1435	1293	142 (9.9%)	
>2 h	458	416	42 (9.2%)	
**Do you eat at least 1.5 bowls of vegetables and two servings of fruit every day**				
No	4146	3504	642 (15.5%)	
Yes	16,329	14,677	1652 (10.1%)	
Unanswered	38,416			
**Whether relatives have had colorectal cancer**	58,401	51,797	6604	0.371
No	55,227	51,797	6604	
Yes	3174	48,966	6261 (11.3%)	
Unanswered	490	2831	343 (10.8%)	
**Whether relatives have had cancer**	32,784	28,959		
No	5015			
Yes	37,799			

**Table 4:** Results of Further Diagnosis of Screening Positive Patients.

Diagnosis	N	%	Age (Years/Old)	OB Data (ng/mL)
Total	3607	100	61.4 ± 7.0	139.3 ± 216.7
Normal	70	1.9	60.1 ± 6.5	105.6 ± 184.4
Hemorrhoid	1129	31.3	60.5 ± 7.0	116.6 ± 194.9
Ulcerative colitis	18	0.5	61.5 ± 6.1	159.6 ± 254.3
Polyps	1935	53.6	62.1 ± 7.1	133.8 ± 206.8
CRC	88	2.4	61.4 ± 6.4	438.7 ± 314.7
Other	367	10.2	61.1 ± 6.8	175.6 ± 217.2

**Table 5:** Logistic regression analysis of the basic data of tested participants.

Item	B	S.E.	Wald	Significance	OR (Odds Ratio)	95% CI
**Gender**						
Male	0.375	0.042	77.931	<0.001	1.455	1.339–1.581
Female (Ref)						
**Age (years/old)**						
50–60	−0.575	0.064	82.011	<0.001	0.563	0.497–0.637
61–70	−0.336	0.057	35.135	<0.001	0.714	0.639–0.798
71–74 (Ref)						
**Education**						
None (Ref)						
Elementary school	−0.078	0.077	1.012	0.314	0.925	0.795–1.077
Junior high school	−0.310	0.088	12.293	<0.001	0.734	0.617–0.872
High school	−0.566	0.089	40.481	<0.001	0.568	0.477–0.676
College, University	−0.549	0.099	30.851	<0.001	0.577	0.476–0.701
Graduate school or above	−0.806	0.181	19.943	<0.001	0.446	0.313–0.636

**Table 6:** Logistic regression analysis of participants’ health behaviors.

Item	B	S.E.	Wald	Significance	OR (Odds Ratio)	95% CI
**Exercise times per weak**						
0–2	−0.024	0.092	0.068	0.795	0.976	0.816–1.168
3–6	−0.047	0.094	0.248	0.618	0.954	0.793–1.148
≧7 (Ref)						
**Average times of exercise per week**						
<30 min	0.205	0.174	1.392	0.238	1.228	0.873–1.726
30–60 min	0.039	0.169	0.053	0.818	1.040	0.746–1.449
1–2 h	0.084	0.185	0.207	0.649	1.088	0.757–1.563
>2 h (Ref)						
**Whether eat at least 1.5 bowls of vegetables and two servings of fruit every day**						
Yes (Ref)						
No	0.355	0.077	21.283	<0.001	1.427	1.227–1.659

**Table 7:** The number of various stages in screening and non-screening group.

	Screening Group (N)	%	Non-Screening Group (N)	%
Pre-cancer	1	1.9	14	2.1
Stage I	11	20.4	75	11.2
Stage II	24	44.4	261	38.8
Stage III	10	18.5	161	24.0
Stage IV	8	14.8	161	23.
Early stage	36	66.7	350	52.2
Late stage	18	33.3	322	47.8
Total	54		672	

## Data Availability

Participants were enrolled from Show Chwan Memorial Hospital. The data presented in our study are available on request from the first and corresponding author. The data are not publicly available due to limitations of obtaining approval from the IRB for the disclosure of data. If anyone requires the data of this study, please to contact the corresponding author.
